# Drug use and opioid substitution treatment for prisoners

**DOI:** 10.1186/1477-7517-7-17

**Published:** 2010-07-19

**Authors:** Heino Stöver, Ingo Ilja Michels

**Affiliations:** 1 Institute of Addiction Research, University of Applied Sciences, Nibelungenplatz 1, D-60318 Frankfurt am Main, Germany; 2 Project Leader of Central Asia Drug Action Programme (CADAP) of the European Union, Deutsche Gesellschaft für Technische Zusammenarbeit (GTZ), Bishkek, Kyrgyzstan

## Abstract

Drug use is prevalent throughout prison populations, and, despite advances in drug treatment programmes for inmates, access to and the quality of these programmes remain substantially poorer than those available for non-incarcerated drug users. Because prisoners may be at greater risk for some of the harms associated with drug use, they deserve therapeutic modalities and attitudes that are at least equal to those available for drug users outside prison. This article discusses drug use by inmates and its associated harms. In addition, this article provides a survey of studies conducted in prisons of opioid substitution therapy (OST), a clinically effective and cost-effective drug treatment strategy. The findings from this overview indicate why treatment efforts for drug users in prison are often poorer than those available for drug users in the non-prison community and demonstrate how the implementation of OST programmes benefits not only prisoners but also prison staff and the community at large. Finally, the article outlines strategies that have been found effective for implementing OST in prisons and offers suggestions for applying these strategies more broadly.

## Introduction: Drug use by prisoners

Drug use remains endemic among incarcerated populations [1,2]. In Europe, the prevalence of drug dependence among prisoners varies from country to country; a systematic review of the literature found the prevalence to range from 10% to 48% for male prisoners and 30% to 60% for female prisoners at the point of incarceration [3]. In the United States, the number of people incarcerated annually for drug-related offenses in the past 20 years has grown from 40,000 to 450,000, leading to prison populations with high rates of drug use [4]. Imprisonment of drug users for crimes they commit--often to support their addiction--contributes to prisoners' high prevalence of drug dependence [5]. A lifetime history of incarceration is common among intravenous drug users (IDUs); 56% to 90% of IDUs have been imprisoned previously [6]. Drug-using prisoners may be continuing a habit acquired before incarceration or may acquire the habit in prison [7,8]. In Europe, 16% to 60% of prisoners who injected outside prison continued to inject while incarcerated [5], whereas 7% to 24% of prisoners who injected said they started in prison [5]. In another study, one-fifth of prisoners injected drugs for the first time in prison [9].

Imprisonment also favours high-risk behaviour regarding drugs because of concentrated at-risk populations and risk-conducive conditions such as overcrowding and violence. The consequences of drug use in prison include drug-related deaths, suicide attempts and self-harm. Drug use tends to be more dangerous inside than outside prisons because of the scarcity of drugs and sterile injecting equipment [5,10,11]. In a study of 492 IDUs, 70.5% reported sharing needles while in prison compared with 45.7% who shared needles in the month before imprisonment (*P *< 0.0001) [9]. Of particular concern is that sharing injecting equipment inside prisons is a primary risk factor for human immunodeficiency virus transmission [12]. Additionally, hepatitis C virus infection through shared injecting equipment in prison has been reported in studies undertaken in Australia [13,14] and Germany [15]. Drug use in prison is also associated with the risk for involvement in violence. Inmates who incur disciplinary action related to possession or use of a controlled substance or contraband were 4.9 times more likely to display violent or disruptive behaviour than those who did not incur such disciplinary action [16]. Prisoners using drugs are also at risk for engaging in further illicit activity [17]. If discovered using illegal drugs, inmates risk prolonged incarceration for breaking security rules and eliciting hostility among prison staff [2,12].

Unless a prisoner receives adequate treatment, drug addiction and dependence and their attendant dangers persist after the prisoner's release into the community and are associated with a high rate of overdose and other harms. Overall, the determining factor in drug-related deaths soon after release appears to be altered tolerance to opioids [18]. In the week after release, prisoners are approximately 40 times more likely to die than are members of the general population; in this immediate post-release period, more than 90% of deaths are drug related [18]. Among women, the odds of a drug-related death in the first week after release were > 10 times greater than at 52 weeks (overall risk [OR] = 10.6; 95% confidence interval [CI] = 4.8-22.0); among men, the odds were ~8 times greater (OR = 8.3; 95% CI = 5.0-13.3) [19]. Very high rates of drug-related mortality persist at least through the first 2 weeks after release from prison [20]. Among the costs to society for an inmate's failure to fully reform while in prison is increased risk for recidivism. Within 12 months of release from prison, 58% of heroin users who did not receive opioid substitution therapy (OST) were re-incarcerated compared with 41% of those who did receive OST [21].

This article provides a non-systematic overview of the literature comparing the quality of drug treatment for inmates with their non-incarcerated counterparts. Guidance regarding the implementation of drug treatment programmes was collected from the literature and included herein. All searches were conducted using Web-based search engines (e.g. PubMed, EMBASE) or abstract archiving system (e.g. SciFinder) combining terms related to incarceration (eg, prison, prisoner) with terms related to drug misuse and treatment (eg, heroin, OST); the end date for searches was December 2009.

## Current state of drug treatment health care efforts for inmates

Many data attest to the low quality or non-existence of drug treatment health care efforts for prisoners compared with efforts made for non-prisoner drug users. For example, in early 2007, 24 of 25 European Union member states had needle exchange programmes in the community, but only three had such programmes in prisons, and only Spain covered all prisons [7]. An international survey reported in 2009 that at least 37 countries offered OST in community settings but not in prison settings [22]. European countries not offering OST in prison include Bulgaria, Cyprus, Estonia, Greece, Latvia, Lithuania, Slovakia and Sweden [22]. OST was considered any treatment for opioid dependence using a medicinal opioid such as methadone, buprenorphine or buprenorphine/naloxone [22]; this differs from methadone maintenance treatment (MMT), in which methadone is the only agent used for substitution therapy.

Universally, the percentage of drug users offered OST varied considerably from prison to prison (from 2% to 16.2%), but utilisation of these programmes was uniformly low (e.g. 7.8% of drug addicts in French prisons received OST) [23]. In most European countries that offered OST in prison, access to and varieties of available OST programmes were heterogeneous and inconsistent [5,24]. For example, although OST is nominally available in German prisons, implementation is the responsibility of each of the 16 federal states and often varies from prison to prison within states [25]. In France, many physicians have been reluctant to initiate OST in prison or even to renew existing buprenorphine or methadone prescriptions for prisoners [26]. If substitution treatment is provided, it is often limited to drug detoxification [5,17]. Furthermore, most efforts to scale up OST in the community have not been carried through to the prison setting [24,27,28].

## Why is drug treatment for prisoners not yet comparable to that available for non-incarcerated drug users?

Several factors affect the extent to which prisons provide OST, including the varied health policies of prisons and the difficulties in employing adequate numbers and quality of prison staff [26]. Some prisoners are prevented from entering an OST programme because of excessively restrictive criteria [22]. For example, in some countries OST is limited to inmates who are serving sentences of a particular length, were in treatment before imprisonment or can confirm that they are enrolled in a post-release treatment programme [22]. In Croatia, OST is restricted to persons aged 25 and older who used illegal drugs for ≥ 10 years and heroin for ≥ 5 years [29]. Other limitations related to OST in prisons include a deficiency of psychological and social support for drug-using prisoners [5] and lack of or limited access to certain OST programmes, such as buprenorphine-based regimens, that may be more suitable for use in prison [27,30].

Several theoretical and functional reasons have resulted in drug treatment for prisoners not having parity with drug users in the community. In particular, some societal misconceptions pervade the medical management of drug dependence. There exists a poor understanding of opioid dependence as a chronic and recurring disease; some clinicians may feel that a hedonistic practice indicates a weakness of character [5,24]. Another widespread but mistaken belief involves the benefits of abstinence for drug users, which leads to the omission of maintenance therapy after detoxification, which in turn leads to reversion to opioid use [31]. In Her Majesty's Prison at Leeds, 43% of prisoners with an illicit opioid habit continued to acquire and use opioids even through the first days of imprisonment and completion of a detoxification regimen [32]. There are also socioeconomic reasons drug-using prisoners, particularly IDUs, do not receive appropriate therapy for their drug problem: they are frequently poor and deprived and, therefore, marginalised [33] and not considered worthy of treatment. These beliefs delay the implementation of OST, as does the common perception that prisons should be "drug-free zones" [5]. Prison authorities may also be concerned that OST undermines their efforts to reduce the drug supply in their institutions (i.e. a black market for drugs) [5,33] and that providing needles is, in effect, placing "weapons" in inmates' hands [26].

## Rationales for drug dependence treatment in prisons

### Benefits for the prisoner

There are many reasons drug-using prisoners should be afforded the same quality of health care regarding drug maintenance treatment--including OST--as is available to non-prisoners [12,34,35]. Primarily, it is appropriate to treat prisoners' drug use so that they will not leave prison in worse health than when they entered [33]. OST is recognised as one of the most effective treatment options for opioid dependence [34]. It can decrease the high cost of opioid dependence to users, their families and society at large by reducing heroin use, associated deaths, HIV-risk behaviours and criminal activity. Substitution maintenance therapy is established as a critical component of community-based approaches in the management of opioid dependence.

Many studies have demonstrated the successful application of OST in prison populations with regard to prisoner-centred and non-prisoner-centred outcomes. Positive prisoner-centred outcomes associated with OST include reduced rates of drug abuse and infectious diseases. Prisoners receiving MMT have shown less drug-injecting [11,36,37] and less risk-taking behaviour (e.g. sharing of syringes) [11,38]. In one study, only 1 of 18 (5.6%) prisoners receiving MMT reported heroin use in the past 30 days compared with 15 of 40 (37.5%) prisoners not receiving MMT (*P *< 0.05) [36]. After 4 months in prison, the rate of illicit use of morphine was 27% for MMT-treated prisoners and 42% for controls (*P *= 0.05) [39]. The use of buprenorphine maintenance therapy in prisons has been based chiefly on results obtained outside prisons [23,25]; however, there is growing experience with buprenorphine in prisons [29]. A group of prisoners receiving buprenorphine reported for their designated post-release treatment programme significantly more often than did a comparison group receiving methadone (48% vs. 14%, respectively; *P *< 0.001) [40]. A 2-year study in Puerto Rico is under way to examine the feasibility of initiating prisoners with histories of heroin addiction on buprenorphine/naloxone before their release to determine the effectiveness of such treatment with regard to post-release treatment entry, reduction in heroin use and reduction in criminal activity at 1 month after release [41].

OST in prison has also been associated with reduced rates of infectious disease. Adequate OST has been associated with reduced risk for HCV infection [39], whereas inadequate MMT--periods of < 5 months in one study, for example--was found to be significantly associated with increased risk for HCV seroconversion (*P *= 0.01) [42]. Prisoners receiving MMT with a daily dose > 60 mg during their whole prison sentence were found to be least likely to inject heroin, share needles and engage in HIV risk-taking behaviour while in prison [38]. In another study, needle-sharing and drug-injecting behaviour decreased significantly among prisoners receiving MMT for > 6 months [43]. Additionally, in Spain, there was a significantly reduced sharing of needles by IDUs in an OST programme (Marco A, 1995, personal communication). OST has also been associated with a reduced risk for prisoner death. In one study, no deaths were recorded while prisoners were enrolled in MMT, whereas 17 prisoners died while not enrolled in MMT, representing an untreated mortality rate of 2.0 per 100 person-years (95% CI, 1.2-3.2) [42]. Finally, prisoners receiving MMT have shown a decrease in serious violent drug charges over time, whereas those not receiving MMT showed an increase [21].

Other positive prisoner-centred outcomes related to OST in prison can be observed after the term of incarceration is completed. Reduced drug use after release was reported among prisoners engaged in an MMT plan [35]. The mean number of days in community-based drug abuse treatment 1 year post-release--as a function of in-prison treatment for drug abuse--was 23.1 days' counselling only in prison; 91.3 days' counselling plus passive transfer to treatment upon release; and 166.0 days' counselling plus methadone treatment in prison and continued post-release (each pairwise comparison, *P *< 0.01). Participants in the counselling-plus-methadone group were significantly less likely than those in the other groups to have opioid-positive or cocaine-positive urine drug test results [44]. OST also lessens the likelihood of released prisoners committing crimes [35]. The reported number of days of criminal activity in the past 365 days after release was 106.7 (standard deviation [SD] = 128.7) with counselling only; 65.2 (SD = 96.2) counselling plus transfer to methadone; and 81.8 (SD = 109.5) days counselling plus methadone [44]. Reduced recidivism was reported among prisoners engaged in some type of OST [45]. Prisoners on a 12-month MMT while incarcerated had a lower level of re-incarceration than heroin-using prisoners with no treatment [21]. Reduced rates of re-incarceration during a 3 1/2-year period following a first incarceration were related to maintenance OST in prison [46]. A Correctional Service of Canada study found that, after 1 year, 41% of addicted inmates receiving MMT were re-admitted to prison compared with 58% of addicted inmates who were not receiving the treatment [46]. Compared with periods of no MMT in prison, the risk for re-incarceration was reduced by 70% during MMT periods ≥ 8 months (*P *< 0.001) [42].

### Benefits for the prison staff and community

A major rationale for the use of OST in prison is the cost-effectiveness of such a strategy. For example, prison methadone is no more costly than community methadone and provides the benefit of reduced heroin use in prisons with the associated reductions in morbidity and mortality [47]. The cost of an institutional OST programme may be offset by the cost savings accruing from offenders successfully remaining in the community longer than equivalent offenders not receiving OST [21,47]. Expanded access to MMT has an incremental cost-effectiveness ratio of < $11,000 per quality-adjusted life-year, which is more cost-effective than many widely used medical therapies [48]. Implementing OST in prisons is also associated with improvement in inmate manageability and prison safety; total institutional charges for prisoners enrolled in MMT are lower than for prisoners not enrolled in MMT [21]. Reduced drug use and reduced recidivism were reported among prisoners engaged in methadone treatment [35].

## Strategies for implementing appropriate maintenance therapy in prisons

Among the more successful strategies for implementing appropriate maintenance therapy in prisons are those used in Spain and the United Kingdom. The mechanics of these programmes may be applicable in other countries that want to implement appropriate maintenance programmes in prisons.

### The Spain model

For more than 10 years, all prisons in Spain have had a legal duty to implement MMT programmes involving syringe exchanges. Incoming prisoners are given a full medical examination, and those who are drug users are offered a treatment programme in which medications are given daily. The laboratory-produced methadone is pre-packaged with the dose for each prisoner in the programme. Prisoners must present identification at the point of medication dispensing and are watched to ensure that the complete dose is taken [49].

### The United Kingdom model

In the United Kingdom, the prison programme (Integrated Drug Treatment System [IDTS]) is funded to provide OST in every adult prison, within an integrated clinical and psychosocial treatment approach, uniting prisons' psychosocial drug treatment services (counselling, assessment, referral, advice and through-care services) and clinical substance misuse management (incorporating the option of MMT or detoxification) services. The design of the programme took into account the vulnerability of drug-using prisoners to suicide and self-harm in prison and to death upon release from prison because of accidental opioid overdose, prison regimen services that correspond to national and international good practice and the need to provide clinical interventions that harmonise with practice in the community and other criminal justice settings.

The United Kingdom programme organised five "work streams" to develop national policies and strategies that would (1) facilitate the integration of the two halves of IDTS; (2) develop a guidance document indicating how IDTS would work with community and criminal justice partners; (3) design and commission a large research study of IDTS; (4) develop a workforce strategy, setting out the knowledge and skills requirements for staff involved in IDTS; and (5) produce a performance management framework, setting out how indicators of performance would be collated.

Training was planned to ensure that staff responsible for the well-being or treatment of IDTS service users had the requisite knowledge and skills for the role. Funding for the United Kingdom programme included a sufficient amount for the purchase and installation in prisons of computer-controlled methadone-dispensing devices. IDTS partners received guidance on staff recruitment, with materials for a national advertising campaign. The creation of shared locations in prisons for IDTS team members and the provision of adequate space for IDTS facilities, including harm minimisation groups and treatment rooms, were actively encouraged. From 2008 to 2009, more than 19,000 MMT treatments were administered in United Kingdom prisons; this number will continue to increase until the full implementation of the IDTS programme occurs in 2010 to 2011.

## Guidance on overcoming barriers to the implementation of substitution programmes in prisons

### Overcoming barriers from the prisoner

Prisoner resistance to participation in a maintenance programme is often based on a lack of desire to be treated. Of 140 eligible men approached to take part in a study of opioid detoxification, 36% declined to be recruited [32]. A similar lack of desire to be treated may be seen with regard to OST. Some prisoners may resist participating in a programme because they do not want their partners or relatives to know they have been using drugs. Some may resist treatment with methadone because they consider methadone a street drug.

Prisoners' refusal to participate in a maintenance programme is best addressed by improving prisoner education. Prisoners may be convinced to participate in a substitution maintenance programme through discussion that includes an explanation and demonstration, through the use of data, of benefits accruing from in-prison OST, including easier incarceration with less desire to inject an illicit drug [17] and the potential for less violence [16], less risk for prolonging incarceration or of irritating prison staff [2], less risk for acquiring an infectious disease [5] and less risk for self-harm. Other benefits that may be demonstrated are realised after release from prison, including less desire to commit crime and, consequently, lower risk for re-incarceration and lower risks for violence, potentially lethal overdose [18] and infectious disease [44].

### Overcoming barriers from the prison staff and other stakeholders

Stakeholders who lack understanding or misunderstand the value of maintenance treatment in prisons--and who may block the implementation of a treatment programme--include politicians, ministerial representatives and prison staff and professionals. A necessary step in convincing stakeholders to support the development of an OST programme is to educate them on the nature of the opioid drug problem among prisoners and on evidence-based benefits of successful OST, including health economics benefits.

Stakeholders need instruction that opioid dependence is a chronically relapsing disease [24] and that coercive abstinence in prison may be followed by relapse immediately after release, often resulting in overdose, drug emergencies and death [19]. This education may include evidence of beneficial results of OST, including reduced rates of drug abuse, both in prison and after release from prison [23,25,35,36,39], less risk-taking behaviour [11,38], reduced rate of infectious disease acquisition [39,42], reduced risk for death [42], decrease in serious violent drug charges [21], reduced criminal activity after release [44] and reduced re-incarceration rate [21,42,45,46]. Outcomes and health economic data demonstrating results of studies showing the cost-effectiveness of drug maintenance therapy in prisons [21,47] should be included. Techniques and resources to gain support for instituting an OST programme and to disseminate information in support of such a programme include initiating and maintaining contact with decision-making politicians, the media, the professional public and non-governmental organisations such as human rights agencies, the United Nations Office on Drugs and Crime and the World Health Organization Regional Office for Europe Health in Prison Project. Other techniques for obtaining and building support for a programme include publishing and making available information on best OST practices; promoting the exchange of knowledge and experience among scientists, politicians and practitioners through international and national conferences of experts from various fields; and organising local and regional discussions among interested physicians. Finally, identifying local "champions" who can knowledgeably explain models of best practice to their peers and provide opportunities for personnel who are interested in starting an OST programme to visit prisons where successful harm reduction programmes are in operation can be invaluable in the process.

Stakeholders should be informed that an OST programme must provide for the supply of OST medications. Lack of access to these medications is often a barrier to the successful implementation of an OST programme. Prisons may have a limited list of medications available for dispensing, and OST maintenance medications may not be among those available. In some cases, there may not be medication available to continue maintenance therapy that was started before imprisonment. Prisoners usually do not have health insurance while in prison and thus cannot afford medication they could afford outside prison; they are dependent for their medications on a prison's health care system.

Prison staffs often express a concern that an OST programme introduces the potential risk for internal diversion of maintenance drugs [17]. In some studies, such diversion was suspected [40], whereas in others it was found not to be a problem [36]. When diversion was suspected, it was because of actions such as movement of a prisoner's hand to the face when sublingual buprenorphine was administered [40]. Because it takes 5 to 10 minutes for a buprenorphine tablet applied sublingually to be absorbed completely, there is time for it to be removed from the mouth after insertion for subsequent potential black-market sale. Prison personnel are often unwilling to spend the time necessary to observe each administered dose of buprenorphine in order to prevent its extraction from the mouth and diversion. Thus, instead of buprenorphine tablets, prisons are increasingly administering tablets combining buprenorphine and naloxone to reduce potential diversion and misuse: applied sublingually, the naloxone is poorly absorbed and has limited pharmacological effect, whereas the efficacy of the buprenorphine is not affected by the presence of naloxone. If a buprenorphine/naloxone tablet is crushed and used intravenously, the naloxone is bioavailable; it will counteract the potential euphoric effect of the buprenorphine and can precipitate severe opioid withdrawal, a strong deterrent to intravenous misuse of diverted buprenorphine/naloxone [28].

Finally, lack of adequate funding to cover start-up costs of a prison OST programme constitutes a barrier to implementing a programme. To remove this barrier, the following items must be covered in a programme's start-up budget: general administration and administration of the OST programme; medical and nursing staffs to execute maintenance therapy assessments, administration and delivery; pharmacy and courier services for stocking, preparation and delivery of medications; disposable materials used in medicating prisoners; maintenance medication; and correction officers to supervise medication administration to prisoners [47].

## Conclusion

Imprisoned IDUs have the basic right to receive treatment for their drug addiction comparable to treatment available to IDUs in the community. This treatment should include OST, a treatment modality with demonstrated broad benefits to prisoners, both while they are incarcerated and after their release from prison, as well as benefits to the community. Examples of successfully implemented OST programmes exist, and these point to effective strategies and tactics for establishing OST programmes elsewhere.

## Competing interests

The authors declare that they have no competing interests.

## Authors' contributions

HS participated in the writing of the manuscript and read and approved the final manuscript. IIM participated in the writing of the manuscript and read and approved the final manuscript.
